# Hemoperitoneum during Pregnancy: A Rare Case of Spontaneous Rupture of the Uterine Artery

**DOI:** 10.1155/2020/8882016

**Published:** 2020-10-08

**Authors:** Catarina Marçal da Silva, Rita Luz, Manuela Almeida, Daniel Pedro, Bárbara Paredes, Rui Branco, Alcides Pereira

**Affiliations:** ^1^Department of Obstetrics and Gynecology, Hospital Garcia de Orta, Almada, Portugal; ^2^Department of Anesthesiology, Hospital Garcia de Orta, Almada, Portugal; ^3^Department of General Surgery, Hospital Garcia de Orta, Almada, Portugal

## Abstract

Spontaneous rupture of the uterine artery is a rare cause of hemoperitoneum during pregnancy. This is a life-threatening condition associated with maternal and fetal mortality. We describe a case of spontaneous rupture of the left uterine artery in a 32-year-old healthy pregnant woman with an uneventful pregnancy.

## 1. Introduction

Hemoperitoneum due to spontaneous rupture of uterine vessels is a rare but life-threating condition during pregnancy [1, 2]. The current maternal mortality was reduced due to improved resuscitative, anesthetic, and operative techniques. Nonetheless, perinatal mortality remains high [2]. When assessing a pregnant patient with a sudden abdominal pain with hemodynamic collapse, the rupture of uterine vessels should be hypothesized and excluded [2, 3].

## 2. Case Presentation

A 32-year-old woman, gravida 1, para 0, presented to our obstetric emergency department at 22 weeks of gestation with severe abdominal pain with a sudden onset after a bowel movement followed by lipothymia. She denied trauma to the abdominal region. She had no previous medical, gynecological, or surgical history, and her antenatal course had been uneventful. At admission, the patient complained of general malaise and worsening abdominal pain. At examination, she was pale and sweaty, blood pressure (BP) was 77/43 mmHg, heart rate (HR) 90-110 bpm, no fever, with normal uterine tonus, and no vaginal bleeding. After immediate fluid resuscitation, the patient remained hypotensive (BP 98/65 mmHg) and tachycardic (HR 110 bpm). Oxygen saturation was normal. The abdomen was distended; there is general tenderness at palpation with abdominal guarding and lack of bowel sounds. There were no signs of vaginal discharge or bleeding. The cervix was long and closed.

**Figure 1:**
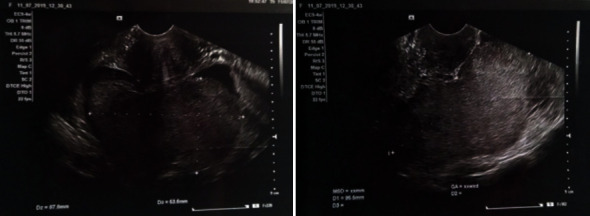
Abdominal Ultrasonography (US) in the emergency room revealed an echogenic mass on the pouch of Douglas.

Abdominal Ultrasonography (US) in the emergency department revealed a normal intrauterine pregnancy with a single alive fetus. On the pouch of Douglas, there was an echogenic image with 95 × 88 × 53 mm suggestive of a blood clot (Figure 1). There was a small amount of free fluid in the perihepatic and perisplenic space.

Blood work at admission showed hemoglobin (Hb) of 10.4 g/dL (first trimester Hb was 12.4 g/dL) with no other abnormalities of the complete blood count, coagulation tests, and hepatic or cardiac enzymology. Arterial blood gas revealed a mildly increased level of lactate (3 mmol/L). General surgery consultation was requested.

An abdominal-pelvic computerized tomography (CT) was performed to aid the differential diagnosis. Immediate evaluation of the CT scan images revealed hemoperitoneum but without an identifiable origin. At this point, the tachycardia was severe (150-160 bpm), and the abdominal pain was worsening. Assuming hemorrhagic shock with hemoperitoneum, the team decided to perform an emergency laparotomy.

The differential diagnosis of a pregnant patient with a sudden abdominal pain with hemodynamic collapse with no external bleeding included uterine rupture, sepsis, aortic dissection, and venous thromboembolism. The echogenic retrouterine image suggesting blood clots with free abdominal fluid, the drop in Hb levels, and tachycardia strongly suggested a hemorrhagic shock with an intra-abdominal origin. In the US, there were no signs of uterine rupture, and the CT scan did not show any signs of contrast extravasation. The origin of the intra-abdominal hemorrhage could have been caused by obstetrical (uterine rupture, rupture of uterine vessels, or rupture of pregnant noncommunicating rudimentary horn) or nonobstetrical (rupture of abdominal aortic aneurysm, rupture of a splenic artery aneurysm/vein, liver rupture-spontaneous, hematoma, hemangioma, or metastasis) conditions [2]. In this clinic case, the diagnosis was made intraoperatively.

**Figure 2:**
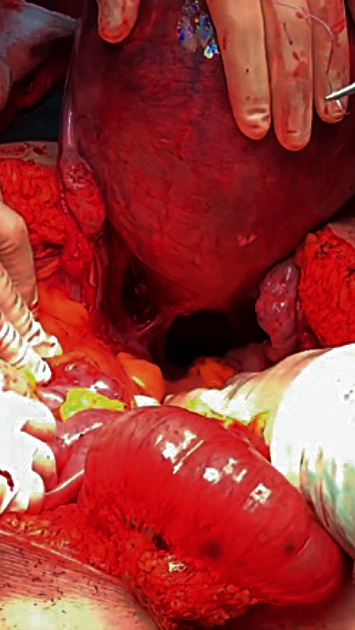
Laceration of the posterior left leaflet of the broad ligament after the first suture on the uterine arterial branch.

Under general anesthesia, an exploratory laparotomy was performed. During the procedure, a laceration of the left posterior leaf of the broad ligament was detected. An active site of bleeding from the left uterine artery branch with blood pulsating from the laceration was identified and sutured (Figure 2). There were no signs of obvious underlying condition such as endometriosis observed. The estimated blood loss was 2000 mL. Intraoperatively, the patient was resuscitated with 1600 mL of crystalloids, 4 units of erythrocyte concentrate, 3 units of fresh frozen plasma, 2 g of fibrinogen, and 1 g of tranexamic acid. The patient was admitted to the intensive care unit under vasopressor support. An abdominal US performed immediately after surgery revealed intrauterine fetal death.

The recovery was favorable, and psychological support was offered. Delivery of the fetus occurred spontaneously on day 4. The patient was discharged on day 6 after admission under lactation suppression.

Fifteen days after admission, the patient became dyspneic and complained of pleuritic chest pain—a pulmonary thromboembolism was diagnosed. Anticoagulation (apixaban) was instituted. Three months after, the patient was stable with no other hemorrhagic/thrombotic events. In the interim, the patient withholds her reproductive life plan, and her main concern is the recurrence of the spontaneous uterine artery laceration. Regarding the possibility of recurrence, a pelvic angio-MRI was performed revealing no further vascular malformations.

## 3. Discussion

Spontaneous rupture of the uterine artery is an extremely rare event. There are only 150 cases described of spontaneous hemoperitoneum in pregnancy secondary to rupture of the uteroovarian vessels or uterine varicose veins, but there is no similar data regarding arterial rupture. The real incidence of this condition is unknown [3, 4].

This diagnosis should be considered when a pregnant patient, especially during the 3^rd^ trimester [1, 2, 5], complains of sudden abdominal pain with hemodynamic collapse along with a decrease in Hb levels. In most of cases, the hemorrhagic site is found on the broad ligament [1]. The outcome is often dramatic with maternal and/or fetal death [2].

The exact etiology of spontaneous rupture of the uterine artery in relation to pregnancy is unknown. Pressure dynamics and anatomic and hormonal factors have been suggested [4]. In this case, there was no history of trauma, endometriosis, or signs that pointed to an inherited connective tissue disorder (for example, vascular Ehlers-Danlos syndrome). There are some questions that remain: the symptoms began after Valsalva maneuver—was the augmented intra-abdominal pressure enough to cause the hemoperitoneum? Was there any vascular uterine malformation?

During the differential diagnosis, if the patient is hemodynamic stable, imaging is useful (US, CT scan with contrast, and angiography), but in most cases, the diagnosis is made intraoperatively [5]. The treatment consists on aggressive resuscitation and laparotomy to control the hemorrhagic sites. Another alternative is the uterine artery angioembolization, taking into consideration the hemodynamic stability of the patient and the fetal gestational age, along with the availability of capable specialists and equipment [4, 5].

The risk of recurrence of spontaneous uterine artery rupture is unknown [4]. There is no evidence in the literature of case reports regarding subsequent pregnancies after spontaneous hemoperitoneum.

## 4. Conclusions

The uterine artery rupture is a life-threating condition that must be suspected and treated promptly. In the event of a sudden abdominal pain with hemodynamic collapse, this diagnosis should be excluded. Currently, the treatment consists in aggressive resuscitation and hemostatic control. The risk of recurrence is unknown.
